# Using Unmanned Aerial Systems (UAS) to assay mangrove estuaries on the Pacific coast of Costa Rica

**DOI:** 10.1371/journal.pone.0217310

**Published:** 2019-06-05

**Authors:** Adam Yaney-Keller, Pilar Santidrián Tomillo, Jordan M. Marshall, Frank V. Paladino

**Affiliations:** 1 Purdue University Fort Wayne, Fort Wayne, Indiana, United States of America; 2 The Leatherback Trust, Goldring-Gund Marine Biology Station, Playa Grande, Costa Rica; Texas Tech University, UNITED STATES

## Abstract

Mangrove forests, one of the world’s most endangered ecosystems, are also some of the most difficult to access. This is especially true along the Pacific coast of Costa Rica, where 99% of the country’s mangroves occur. Unmanned Aerial Systems (UAS), or drones, have become a convenient tool for natural area assessment, and offer a solution to the problems of remote mangrove monitoring. This study is the first to use UAS to analyze the structure of a mangrove forests within Central America. Our goals were to (1) determine the forest structure of two estuaries in northwestern Costa Rica through traditional ground measurements, (2) assess the accuracy of UAS measurements of canopy height and percent coverage and (3) determine whether the normalized difference vegetation index (NDVI) could discriminate between the most abundant mangrove species. We flew a UAS equipped with a single NDVI sensor during the peak wet (Sept–Nov) and dry (Jan–Feb) seasons. The structure and species composition of the estuaries showed a possible transition between the wet mangroves of southern Costa Rica and the drier northern mangroves. UAS-derived measurements at 100 cm/pixel resolution of percent canopy coverage and maximum and mean canopy height were not statistically different from ground measurements (p > 0.05). However, there were differences in mean canopy height at 10 cm/pixel resolution (p = 0.043), indicating diminished returns in accuracy as resolution becomes extremely fine. Mean NDVI values of *Avicennia germinans* (most abundant species) changed significantly between seasons (p < 0.001). Mean NDVI of *Rhizophora racemosa* (second most abundant species) was significantly different from *A*. *germinans* and dry forest dominant plots during the dry season (p < 0.001), demonstrating NDVI’s capability of discriminating mangrove species. This study provides the first structural assessment of the studied estuaries and a framework for future studies of mangroves using UAS.

## Introduction

Mangrove forests are among one of the most rapidly disappearing ecosystems on earth, declining at a rate of 1–2% per year globally, a rate greater than or equal to declines in adjacent coral reefs and tropical rainforests [1]. Costa Rica alone contains 8% of the world’s mangrove forests, 99% of which are found on the Pacific coast [2]. These forests can provide a number of ecologically important services, including sequestering large amounts of carbon [3, 4], serving as nurseries for many marine fish and invertebrates, including economically important species [2, 4], and providing important stopover sites for migrating Nearctic birds [2, 5]. In areas with distinct dry seasons, such as the north Pacific coast of Costa Rica, these forests may also be important nesting and hunting habitats for a host of different wildlife species, including those displaced by human development in other coastal areas [2, 6]. Despite being protected in Costa Rica under the Coastal-Maritime Law (1977), Wildlife Law (1992) and Environmental Law (1995), mangrove forests in the country are increasingly under threat as human coastal populations grow [2, 7].

Located just south of Santa Rosa National Park, the coastal region of the gulf of Papagayo is an area defined by several relatively pristine mangrove estuaries, fragments of tropical dry forest, and a matrix of individual ranch and farm land holdings. Within this area lie two remote and fragmented mangrove estuaries: Cabuyal and Zapotillal, both of which are understudied [7]. Isolated forests such as these often have low priority in conservation enforcement efforts, and may be eventually developed before they can be accurately assessed. However, recent advances in ecological monitoring technologies, specifically the adoption of Unmanned Aerial Systems (UAS) for aerial survey and mapping, has reduced the time and cost of ecological monitoring to make substantial study of low priority habitats a reality. UAS have presented a cost-effective and relatively simple solution to the problems of obtaining aerial data for ecological purposes, with flight planning software that allows aerial surveys, images, and videos to be obtained with little effort by remote operators [8, 9, 10]. Additionally, mangrove forests and other wetlands present areas of high potential for UAS-based surveys, as they remain difficult to access and survey through traditional ground based methods [11, 12, 13, 14, 15, 16, 17].

One of the most exciting applications of UAS is the creation of both two-dimensional orthomosaic and three-dimensional models of mapped areas via a photogrammetric technique called “structure from motion” (SfM), a method that derives the three-dimensional position of points in overlapping aerial images, creating dense 3D point clouds of a mapped area in a separate image space [18, 19, 20, 21]. From these point clouds measurements of real-world attributes such as topography and forest structure can be made [20, 22, 23, 24]. The addition of other sensors to the UAS can also be used to simultaneously measure plant health, identify vegetation types, and differentiate between land cover types [25, 26, 27, 28]. One such measurement is the Normalized Difference Vegeation Index (NDVI), which measures the differences between near-infrared and red spectral reflectance, generating a value between -1 (i.e. water) and +1 (i.e. healthy green vegetation) to differentiate vegetation from non-vegeation when applied to images of land cover [29, 30, 31]. In remote sensing studies of mangroves, NDVI has been shown to be an accurate method of measuring canopy closure [31], leaf area index (LAI) [32], and species distribution [33].

When combined with image classification techniques, such as Object-Based Image Analysis (OBIA) mangrove cover [11, 34] and with additional spectral bands mangrove species distributions [12, 28] can be classified within images. Recently, the supervised OBIA method of Support Vector Machines (SVM) has been shown to be a robust and accurate method for high-definition UAS imagery classification [12, 28, 35].

The goal of this study was to answer several key questions regarding both the mangrove forests of Cabuyal and Zapotillal and the use of UAS for mangrove surveys. We wanted first to determine the overstory structure of the Cabuyal and Zapotillal estuaries, and whether UAS imagery analysis was an accurate technique for studying canopy height and canopy coverage when compared to traditional ground-based methodology. We also wanted to determine whether there were measurable changes in the wet and dry season NDVI for local mangrove species, using and if a commercial NDVI sensor was a useful tool for determining species distributions within mangrove estuaries.

## Materials and methods

To answer these three questions, we used a small UAS equipped with an NDVI sensor to create georeferenced orthomosaic and three-dimensional maps of the Cabuyal and Zapotillal estuaries during both the wet and dry seasons.

### Site description

The Cabuyal estuary in the Nacascolo district of Guanacaste, Costa Rica (10.66N, 85.64W) is an approximately 60-hectare intertidal estuary characterized by mangrove swamp surrounded by tropical dry forest and several land holdings [36]. The area receives approximately 1400 mm of rain fall a year, almost entirely during the wet season (May–November) and is fed by a seasonal tributary of the Tempisque known as the *Quebrada Cacao*. A description of the site was published in 1998 under the National Wetland Inventory [36], and listed the dominant mangrove species as *Avicennia germinans* (L.) L., *A*. *bicolor* Standl., *Rhizophora mangle* L. and *R*. *racemose* G.Mey. Recent surveys have also confirmed the presence of *Pellicieria rhizophorae* Planch & Triana, a species listed as vulnerable by the International Union for the Conservation of Nature (IUCN) [37]. A much smaller (approximately 1.75 ha), undescribed intertidal estuary known locally as Zapotillal sits approximately one kilometer to the south of Cabuyal, on the Peninsula Papagayo (10.658N, 85.670W). The site is known to contain several species of mangrove and mangrove associate species, including *Laguncularia racemosa* (L.) C.F.Gaertn, *Conocarpus erectus* L. (per. Observation), *A*. *germinans* and *R*. *racemosa*. These sites were chosen for this study due to their relatively small size, minimal development and human interaction, remote location, and conservation importance.

### Ground data acquisition

**Fig 1:**
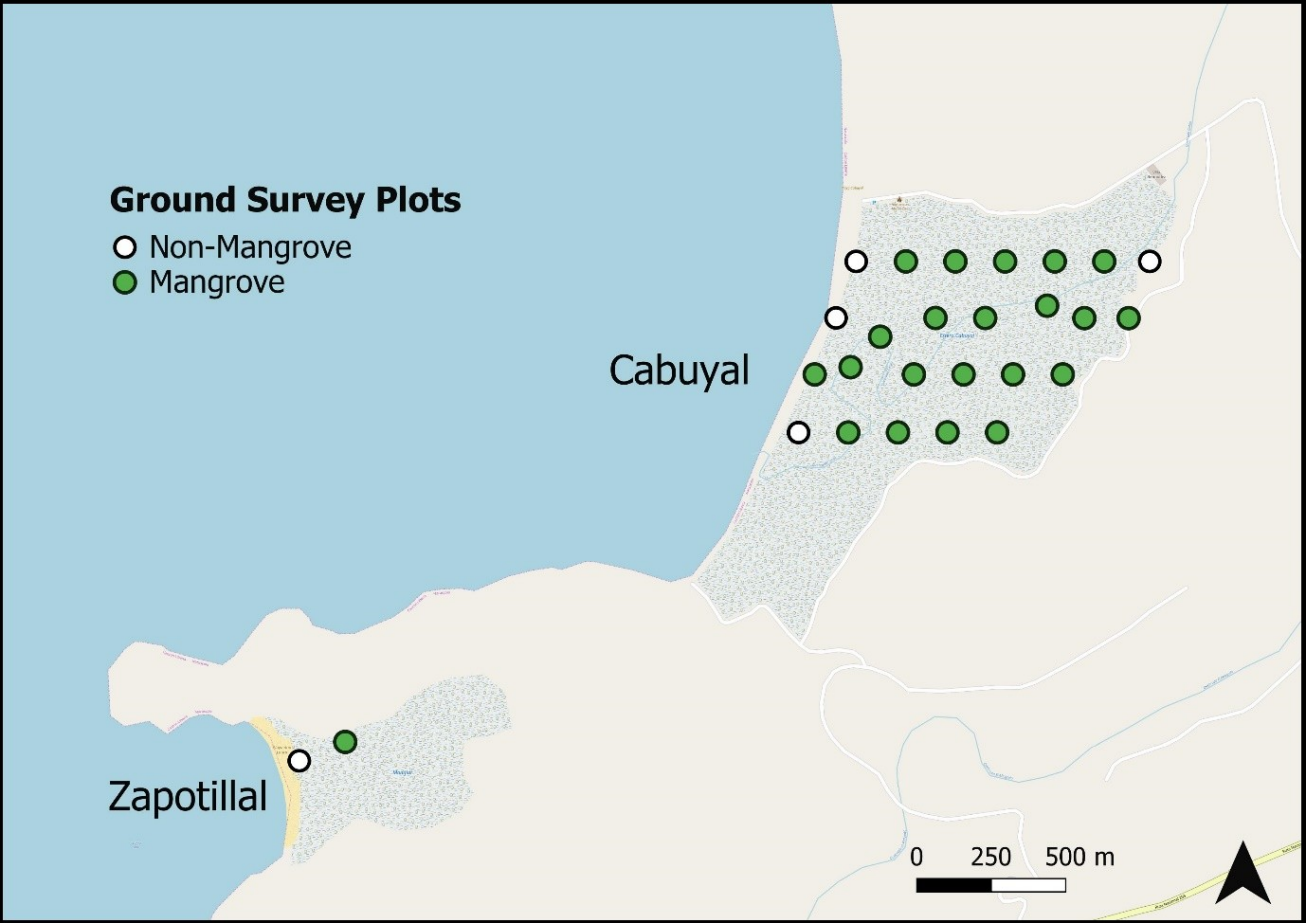
Map of ground survey plot locations within the Cabuyal and Zapotillal estuaries. Plots classified as containing or not containing mangroves or associate species. Map data copyrighted OpenStreetMap contributors and reprinted under a CC BY-SA 2.0 license and available from https://www.openstreetmap.org.

To assess the accuracy of UAS derived forest structure information, traditional on-the-ground methods of data acquisition were employed in both estuaries. Forest structure information was collected between January and February 2018. Four parallel 1 km straight-line transects in the Cabuyal estuary and one 1 km line transect in the Zapotillal estuary were created beginning at the approximate start of vegetation on the seaward edge of the estuary and running inland. Transects in Cabuyal were spaced 200 m from each other and began approximately 200 m from the road which defines the northern edge of the estuary. The transect in Zapotillal was placed at the approximate center of the estuary. Along each transect seven 7 m radius circular plots were created at equivalent spacing. Plots were navigated to using a handheld GPSMap64 (Garmin International, Inc., Olathe, KS) on foot or by kayak. If a plot outside of the estuary did not contain mangroves or associates, it was removed from the study. If a plot inside of the estuary was inaccessible, a new plot was created at the nearest possible accessible point. All GPS positions were determined within ± 5.0 m accuracy, with a ± 3.0 m minimum. In total 27 plots were surveyed (n = 25, Cabuyal and n = 2, Zapotillal), 22 of which contained mangroves or the mangrove associate, *C*. *erectus* (Fig 1).

At the center of each plot, a handheld spherical densiometer was used to measure canopy coverage following Lemmon (1956) [38]. Mangrove and *C*. *erectus* trees within plots were identified to species using the dichotomous keys in Tomlinson (1986) [39], and each tree’s diameter-at-breast-height (DBH, at 1.37 m) was measured. For stilt-root species, diameter was measured at the point above the highest accessible stilt-root, following Kauffman & Donato (2012) [40]. Only mangroves or associates with a DBH greater than or equal to 5 cm were considered overstory and included in the analysis. Canopy height was measured using an Abney hand level (clinometer) as described in Anderson et al. (2006) [41]. When direct measurement of canopy height was not possible, it was estimated by a single observer consistent between plots. Temporary markings were used to ensure the same tree was not measured twice. Mean height, maximum height and percent canopy cover were then calculated for each plot.

The importance value index for each species was calculated as the sum of relative density, relative dominance and relative frequency following Curtis & McIntosh (1951) [42]. Relative density was the number of all individuals of each species as percentage of the total number of individuals, relative dominance was total basal area of each species as a percentage of the total basal area of all species, and relative frequency was the number of plots each species occurs as a percentage of all plots [42]. Above-ground and below-ground biomass was calculated for each tree based on species-specific allometric equations provided by Kauffman & Donato (2012) [40], Komiyama et al. (2008) [43], and Gross et al. (2014) [44]. A dominant species for each plot was determined if it had an above-ground biomass of greater than 90% of the total plot above-ground biomass. If the dominant species in the plot was not a mangrove or associate, the plot was categorized as ‘non-mangrove’.

### UAS image acquisition

UAS data was acquired using a DJI Phantom 3 Professional quadcopter (DJI Inc., Shenzhen, China) mounted with a Sentera high precision NDVI+ single sensor (Sentera, LLC, Minneapolis, MN). The NDVI+ is a commercial precision agriculture sensor which captures images in the red band at 625 nm central wavelength (CWL) x 100 nm width, and the NIR band at 850 nm CWL x 400 nm width. While the NDVI+ sensor is not a strictly dedicated multi-spectral camera (such as the Parrot Sequioa), it uses in-built filtering software to avoid wavelength contamination from other spectral bands by narrowing the band capture in the Red and NIR bands, allowing for an accurate measurement of NDVI at reduced cost. A Sentera Incident Light Sensor was also mounted and integrated with the NDVI+ sensor to correct for changes in ambient light conditions and allow comparable measurements of NDVI between flights and images.

Mapping was conducted during two periods; the late wet season that included the two rainiest months (Sept-Oct) and transition month (Nov) in 2017, and the middle dry season (Jan-Feb) in 2018. Grid-based flight plans were created and executed using the MapPilot application (DronesMadeEasy, Inc., San Diego, CA). All flights were conducted (1) at a height of 85 m, corrected for the altitude at launch, following Cruzan et al. (2016) [45], (2) at a level of 86% front and side overlap and (3) between 90 minutes before and after local solar noon to maintain similar incident light levels between flights. We aborted flights when winds were strong or cloud cover-reached over 80% during a survey. Determining total area surveyed during flights is difficult when using overlapping drone images, however by overlapping the flight programs we are confident that the surveyed area consisted of the entire 60 ha Cabuyal estuary and 1.75 ha Zapotillal estuary.

To increase geolocation accuracy during post-processing, thirteen ground control points (GCPs) in Cabuyal and one in Zapotillal were created within and along the edges of the estuaries in open areas using large natural markers painted white. Horizontal GPS locations of these markers was taken with a handheld Garmin GPSMap64 at ± 3.0 m accuracy.

### Image processing

**Fig 2:**
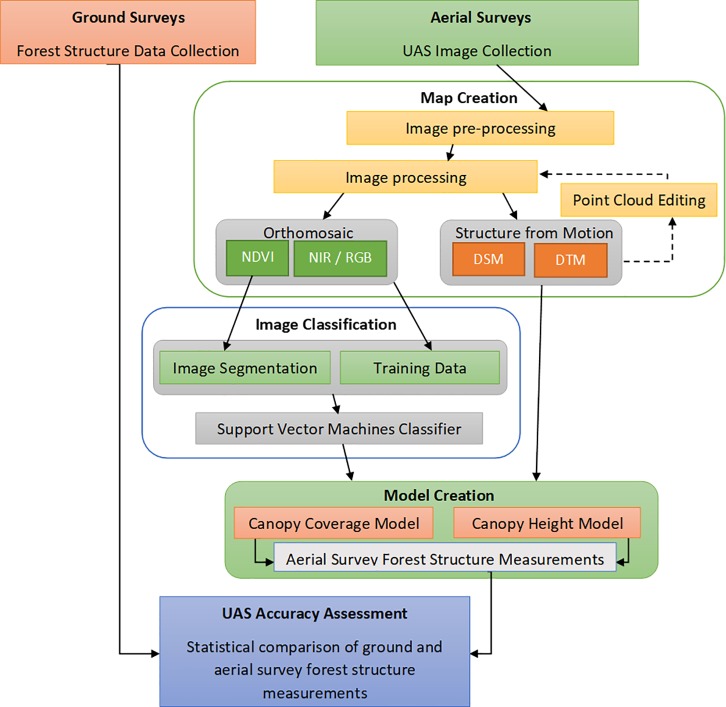
Model workflow of the accuracy assessment of ground data and aerial survey forest structure measurements. Data on forest structure collected from ground surveys was compared to forest structure data derived from canopy height (CHM) and canopy cover (CCM) models applied to support vector machine (SVM) classified UAS imagery maps.

UAS images were processed for data collection and accuracy assessment (Fig 2). NDVI+ sensor images were first uploaded into Sentera FieldAgent software for organization and calibration. Once the desired map areas were delineated within the FieldAgent software, photos from that area were uploaded into Pix4D Mapper software (Pix4D, Inc., Luasanne, Switzerland) to create an orthomosaic map, NDVI index orthomosaic map, digital surface model (DSM) and digital terrain model (DTM) of each survey area. The software utilized the SfM technique to create and classify a dense point cloud from the images. Point-clouds were then manually edited to remove extreme outliers and water features, re-classified into vegetation, ground and building classes, and re-processed into DSMs and DTMs to improve accuracy [20, 46, 47]. Parameters for the creation of the NDVI index within Pix4D Mapper were based on a software template provided by Sentera. Ground control points were identified within the maps and their horizontal GPS location uploaded when present in survey areas. Survey areas were delineated to maximize inclusion of ground-control points, and at least one was included in all map creations. Resolution was manually set at 10 cm and 100 cm per pixel resolution for all dry season maps to allow for simple and accurate comparison between map areas. This was done in order to do a post-hoc test of the effects of capturing images at different altitudes and investigate whether ultra-high or slightly lower resolution images could produce similar results in regards to forest structure analysis. Wet season maps were created at only 10 cm/pixel resolution. Over the Zapotillal estuary, NDVI+ photos were only acquired during the dry season due to a mechanical malfunction in November 2017, which interrupted NDVI+ photography until January 2018. DSMs were processed at the same resolution as orthomosaics, and DTMs were processed at five times pixel resolution as recommended by Unger et al. (2009) [47], except for 100 cm maps, where DTMs and DSMs were processed at the same resolution.

### Image classification

Orthomosaics, DSMs, and DTMs were loaded as georeferenced TIFF files into ArcGIS 10.5 (ESRI, Inc., Redlands, CA) for object-based image analysis (OBIA) classification and geospatial analysis. TIFF files were automatically converted to single-band rasters within ArcGIS and pyramids were built using a bilinear interpolation method without data compression. The NDVI raster were segmented with a high level of spectral and spatial detail. Thirty circular training samples of roughly equivalent size and spread throughout the map were created for vegetation and non-vegetation land-cover classes. Training samples for land cover were distributed throughout the land cover types present; vegetation, water, shadows, buildings, roads, mud, salt flats, sand and canopy gaps. The number of training samples per type were approximated to the percentage of land-cover occupied by that type in each map using visual approximation. Training samples were saved and uploaded along with the segmented raster into the Support Vector Machine classifier which was then used to classify the NDVI orthomosaic raster. All segment attributes were selected for raster classification. No accuracy assessment was conducted.

### Canopy height & canopy cover models

Once the rasters were classified into vegetation and non-vegetation land-cover, they were loaded into a canopy height (CHM) and canopy coverage (CCM) model, which determined mean and maximum canopy height, percent canopy coverage, and mean NDVI values for the 27 ground survey plots.

The CHM began by subtracting the DTM from the DSM, to correct for the effects of variable terrain height in the creation of the DSM [26]. Mean and maximum height of all vegetation pixels above 2.63 m (the lowest tree height measured in the field with DBH ≥ 5 cm) within a 7 m radius circle around the center GPS point of a ground survey plot were then extracted. If the CHM yielded mean and maximum heights below the 2.63 m threshold, it was included in this analysis as repressed mangrove with a minimum height threshold of 1.0 m.

The CCM used the initial step of the CHM to create a raster of vegetative cover above 1.5 m in a 7.5 m radius circular plot around the center of the ground survey plot, allowing for removal of understory vegetation not likely visible by the UAS [48]. This radius represented an area of approximately 10 percent of the total ground sampling distance (GSD) of the sensor at 10 cm/pixel resolution. A raster was created representing vegetation cover within the plot, and the area of that raster was calculated and divided by the total area of the 7.5 m radius plot to determine percentage canopy cover. The mean NDVI value for each plot was then extracted from the total vegetation cover for each plot for 10 cm/pixel resolution maps.

### UAS accuracy assessment

Assessment of the accuracy of structural characteristics derived from UAS imagery was performed via paired statistical analysis of ground-validated and UAS-derived data. Comparisons of the performance of the CHM and CCM were only carried out on dry season map only, due to the lack of wet season imagery available owing to the mechanical failure in November 2017. We tested for normality using Shapiro-Wilks. We used two-tailed paired t-tests when data were normally distributed, and Wilcoxon signed rank test [49] when they were not. We used one-way ANOVAs with Tukey HSD post-hoc to compare NDVI values of dominant species within the wet and dry seasons and two-tailed paired t-tests to compare each plot dominant species’ mean NDVI values between wet and dry seasons. A limited sample set consisting only of plots sampled during both the wet and dry seasons was used for this analysis, and only at 10cm/pixel resolution. Only true mangroves or non-mangrove dominant plots were used for this analysis. All statistical tests were conducted with α = 0.05 in R version 3.5.0 using base packages [50]. The root mean square error (RMSE) is a widely used statistical metric for comparing paired data-sets and evaluating model performance [51, 52], and was calculated for all CHM and CCM results to compare model performance. Paired-differences between ground survey and UAS measurements were defined as residuals, and residuals values were plotted against ground survey measurements to observe patterns in model performance.

## Results

### Ground survey

Twenty-five ground plots in Cabuyal and two ground plots in Zapotillal were surveyed. Among those, 21 plots in Cabuyal and one plot in Zapotillal contained mangroves or associates. Four species of mangroves and were encountered at Cabuyal: *A*. *germinans*, *L*. *racemosa*, *P*. *rhizophorae*, *R*. *racemosa* (Table 1). One species of mangrove associate (*C*. *erectus)* was found in Cabuyal, though it was found outside of the survey area in Zapotillal (Table 1). In comparison, only *L*. *racemosa* and *R*. *racemosa* were encountered within the mangrove plot at Zapotillal (Table 1). The most numerous species were *A*. *germinans* followed by *R*. *racemosa* in Cabuyal and *L*. *racemosa* in Zapotillal (Table 1). Only one individual of vulnerable *P*. *rhizophorae* was occurred within plots in Cabuyal (Table 1), however, a relatively large number of individuals (> 30 trees) of this species were encountered outside of the study transects. *R*. *racemosa* had the greatest mean aboveground biomass of any species in the Cabuyal estuary, however, *L*. *racemosa* within the Zapotillal estuary had the greatest mean aboveground biomass for either estuary (Table 1).

**Table 1:** Number of individuals, mean, minimum & maximum tree height, diameter at breast height (DBH), and total above-ground (ABG) and belowground (BGB) biomass for 259 mangrove and mangrove associate (*C*. *erectus)* trees (≥5 cm DBH) encountered in 21 plots in the Cabuyal and 1 plot in the Zapotillal mangrove stands.

Species		Site	Number of individuals			Mean height ± S.E. (m)	Min.—Max. Height (m)			Mean DBH ± S.E. (cm)	Min.—Max. DBH (cm)			Mean ABG (kg)	Mean BGB (kg)	
*Conocarpus erectus*	Cabuyal			2	5.71 ± 0.20			5.51–5.90	14.25 ± 3.25			11–17.5	86.06			55.59
*Avicennia germinans*	Cabuyal			169	7.02 ± 0.18			2.63–12.5	10.19 ± 0.38			2.6–33.1	52.13			31.43
*Laguncularia racemosa*	Cabuyal			2	10.87 ± 0.92			8.66–13.08	17.80 ± 3.89			15–27	240.31			77.56
*Pelliciera rhizophorae*	Cabuyal			1	6.78			6.78	10.90			10.9	30.75			30.86
*Rhizophora racemosa*	Cabuyal			81	11.98 ± 0.59			3.00–26.11	16.70 ± 1.18			5.4–47.3	382.04			144.78
*Laguncularia racemosa*	Zapotillal			3	19.33 ± 0.67			18.0–20.0	32.23 ± 2.46			28.8–37.0	1096.44			284.88
*Rhizophora racemosa*	Zapotillal			1	3.00			3.00	5.4			5.4	10.28			7.50

In the Cabuyal estuary, *R*. *racemosa* had the highest relative dominance among the five species detected, while *A*. *germinans* had the highest relative density, frequency and importance value index (Table 2). However, *R*. *racemosa* had the highest mean and maximum height, and *R*. *racemosa* had the largest DBH, overlapping with *L*. *racemosa* by standard error (Table 1). *L*. *racemosa* had the highest importance value within the Zapotillal estuary (Table 2).

**Table 2:** Species frequency, tree density, mean basal area and relative density, dominance, frequency and importance value indices for ≥ 5 cm diameter at breast height (DBH) mangrove and mangrove associate (*C*. *erectus)* trees found in 21 plots in the Cabuyal and 1 plot in the Zapotillal mangrove stands.

Species	Site	Species Frequency	Tree Density (t ha^-1^)	Mean Basal Area (m^2^)	Relative Density	Relative Dominance	Relative Frequency	Importance Value Index
***Conocarpus erectus***	Cabuyal	1	14.67	0.02	0.78	0.78	4.76	6.32
***Avicennia germinans***	Cabuyal	11	1239.33	0.01	66.27	39.60	52.38	158.25
***Laguncularia racemosa***	Cabuyal	1	14.67	0.03	0.78	1.19	4.76	6.73
***Pelliciera rhizophorae***	Cabuyal	1	7.33	0.01	0.39	0.22	4.76	5.37
***Rhizophora racemosa***	Cabuyal	10	594	0.03	31.76	58.21	47.62	137.59
***Laguncularia racemosa***	Zapotillal	1	22	0.16	75.0	99.08	100.00	274.08
***Rhizophora racemosa***	Zapotillal	1	7.33	0.03	25.0	0.92	100.00	125.92

### UAS-imagery map creation

**Fig 3:**
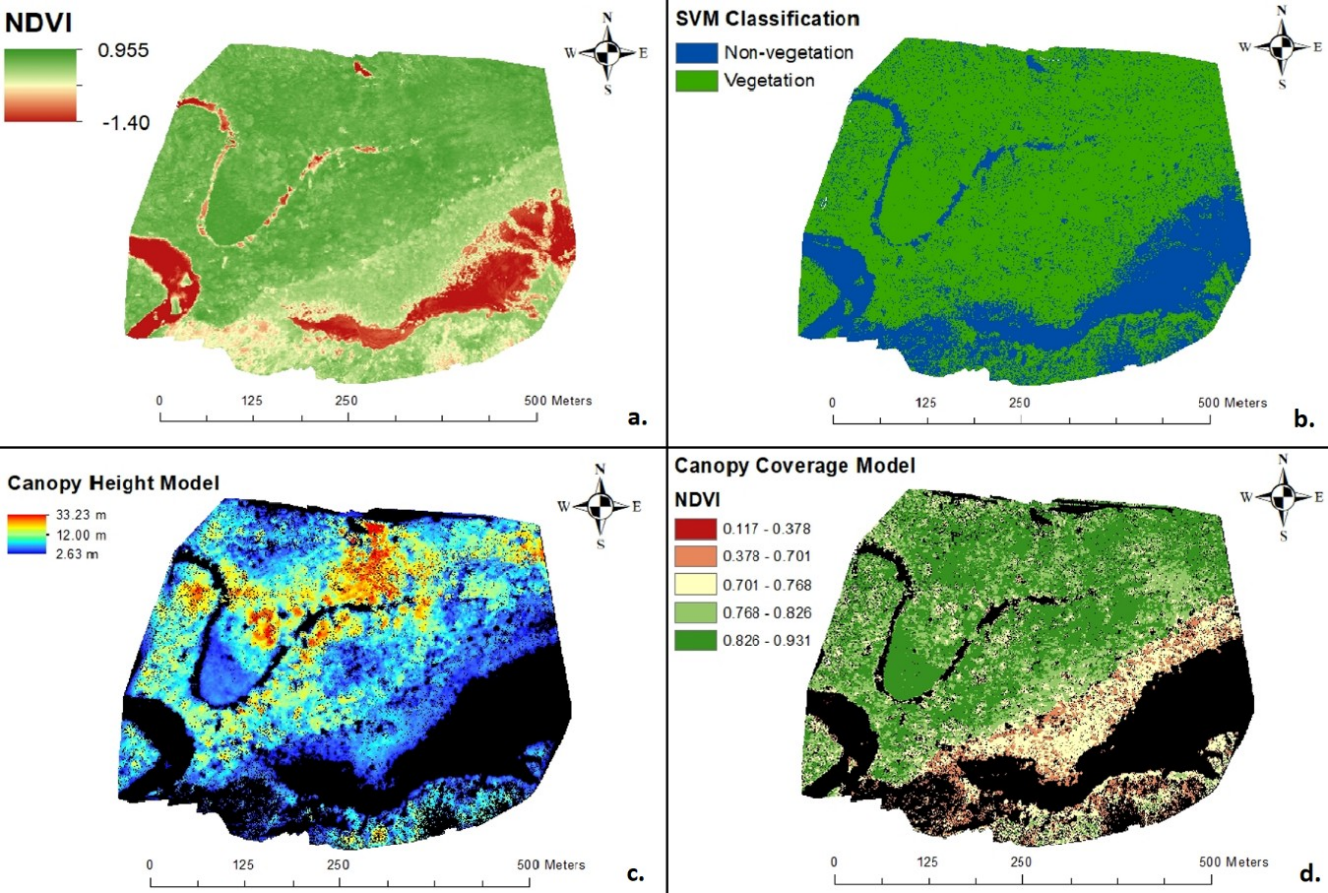
Maps of a southwest 500m x 500m portion of the Cabuyal estuary created from UAS imagery. (A) NDVI index map, (B) support vector machine (SVM) classification of non-vegetation and vegetation land cover, (C) canopy height model of vegetation land cover based on SVM, and (D) canopy coverage model of vegetation land cover based on SVM overlaid with natural jenks break NDVI classification.

A total of 41 drone flights were conducted. Mean flight time was 13 minutes, with a mean of 4.21 km traversed per flight. Over the Cabuyal estuary, 5,271 NDVI+ photos were acquired during the wet season, and 10,373 NDVI+ photos during the dry season. Images captured during the wet season were used to create five 10 cm/pixel, while those captured during the dry season created eight 10 cm/pixel resolution maps and seven 100 cm/pixel resolution maps across both estuaries (Fig 3). Mean absolute geolocation variance for all maps, expressed as root mean square error (m), were between 0.70 and 2.01 m in the x, y, and z directions (Table 3).

**Table 3:** Root mean square (RMS) error in the x, y, and z directions for absolute geolocation variance for photo geolocation and calibration for wet (10cm/pixel resolution), dry (10cm/pixel resolution) and dry (100cm/pixel resolution) maps. Maps created in and geolocation information provided by Pix4D (Pix4D, Inc., Lausanne, Switzerland).

Maps	Mean RMS Error X (m)	Mean RMS Error Y (m)	Mean RMS Error Z (m)
**Wet 10cm**	1.68	0.71	1.31
**Dry 10cm**	1.12	1.07	1.70
**Dry 100cm**	1.23	1.21	2.01

### UAS-imagery–ground survey comparison

Shapiro-Wilks tests revealed that measures of maximum & mean canopy height for 10 cm and 100 cm maps met the assumption of normal distributions, except for mean canopy height UAS measurements for 10 cm maps (W = 0.861, p = 0.005). None of the measures of percent canopy cover met assumptions of normality, and so a Wilcoxon Signed Rank test was used for comparisons. For 10 cm/pixel resolution maps of dry season imagery, paired t-tests indicated no statistically significant difference between UAS-imagery derived (mean = 12.29, SE = 1.54) and ground survey measurements (mean = 12.80, SE = 1.37) of maximum canopy height (t_(2)_ = -0.63, df = 21, p = 0.536). For mean canopy height, the Wilcoxon signed-rank test indicated there was a statistically significant difference between 10 cm UAS (mean = 8.26, SE = 1.13) and ground (mean = 9.19, SE = 0.94) measurements of mean canopy heights (T = 189, df = 22, p = 0.043). The Wilcoxon signed-rank test also showed no significant difference in percent canopy cover measurements (T = 165, df = 27, p = 0.578) from both ground survey (median = 92.72, SD = 25.94, min. = 0.16, max. = 100) and UAS-imagery (median = 94.51, SD = 28.73, min. = 5.07, max. = 100).

For 100 cm/pixel resolution maps of dry season imagery, paired t-tests indicated no statistically significant differences between UAS-imagery derived measurements and ground survey measurements of maximum canopy height (t_(2)_ = 0.75, df = 21, p = 0.462), nor of mean canopy height (t_(2)_ = 0.65, df = 22, p = 0.526). Wilcoxon signed-rank tests indicated that there was no statistically significant difference between 100 cm/pixel UAS and ground measurements of percent canopy cover (T = 137, df = 27, p = 0.219).

**Fig 4:**
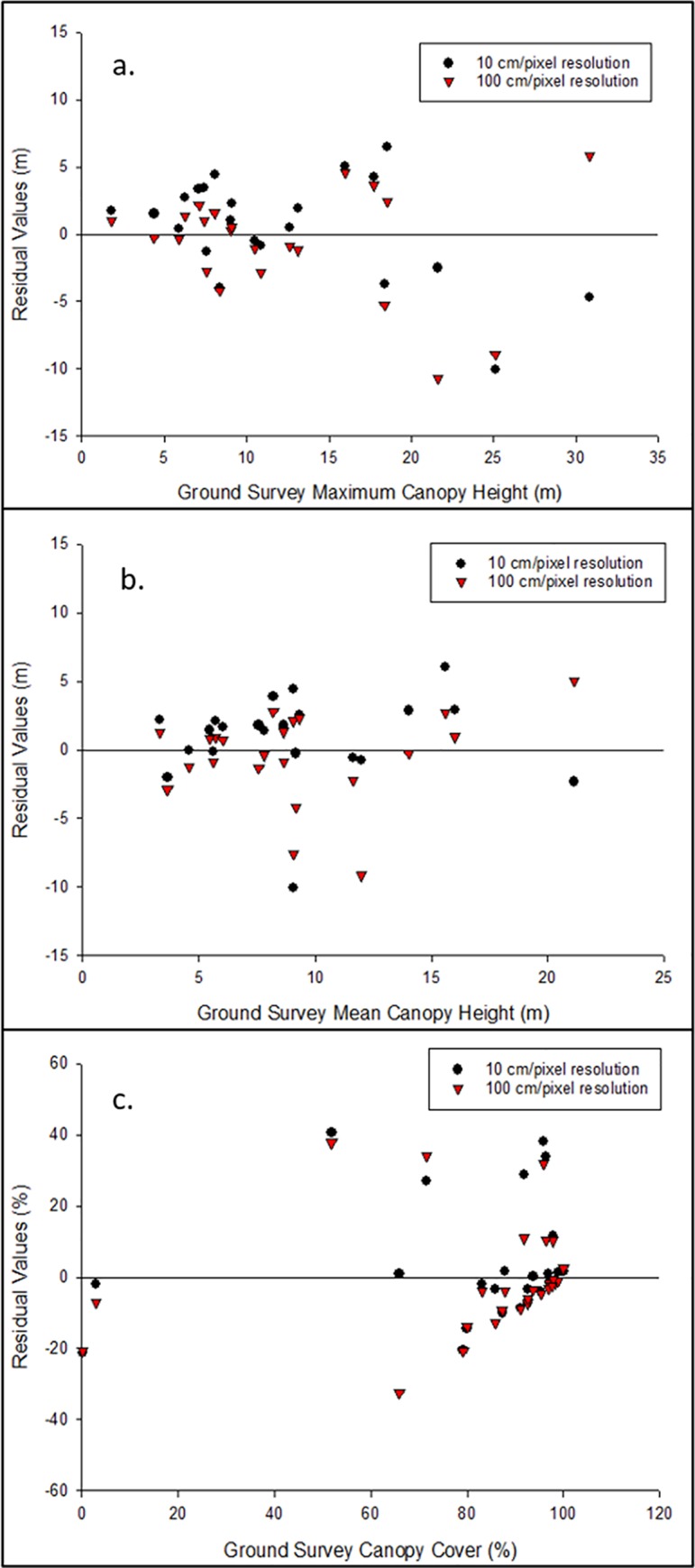
Residuals of UAS and ground measurements of forest structure. These include (A) maximum and (B) mean canopy height (m) and (C) percent canopy cover for 10 cm/pixel and 100 cm/pixel resolution dry season maps of the Cabuyal and Zapotillal estuaries.

RMSE values were lower for both maximum canopy height and percent canopy cover for 100 cm resolution maps than for 10 cm maps (Table 4). However, the RMSE value for mean canopy height was lower for 10 cm maps (Table 4). Residual plots of maximum canopy height (m) showed a general trend of underestimation of taller tree heights for both 10 cm and 100 cm (Fig 4A). Mean canopy height (m) residual plots showed no general trend of either underestimation or overestimation at either resolution (Fig 4B). RMSE was lower for 100 cm resolution CCMs than for 10 cm CCMs, although the difference was low enough to indicate no real difference in model performance (Table 4). Analysis of the residual plot for both 10 cm and 100cm CCM results revealed increasing accuracy in model performance as canopy cover increases (Fig 4C).

**Table 4:** Root mean square errors (RMSE) for 10cm and 100cm UAS imagery derived measurements of maximum and mean canopy heights (n = 22) and percent canopy (n = 27) in the Cabuyal and Zapotillal estuaries. UAS imagery and field measurements collected between January and February 2018.

Observed (Field) v. Predicted (UAS) Measures	RMSE 10 cm	RMSE 100 cm
**Maximum Canopy Height**	3.77m	3.60m
**Mean Canopy Height**	3.23m	3.26m
**Percent Canopy Cover**	16.43%	15.65%

**Fig 5:**
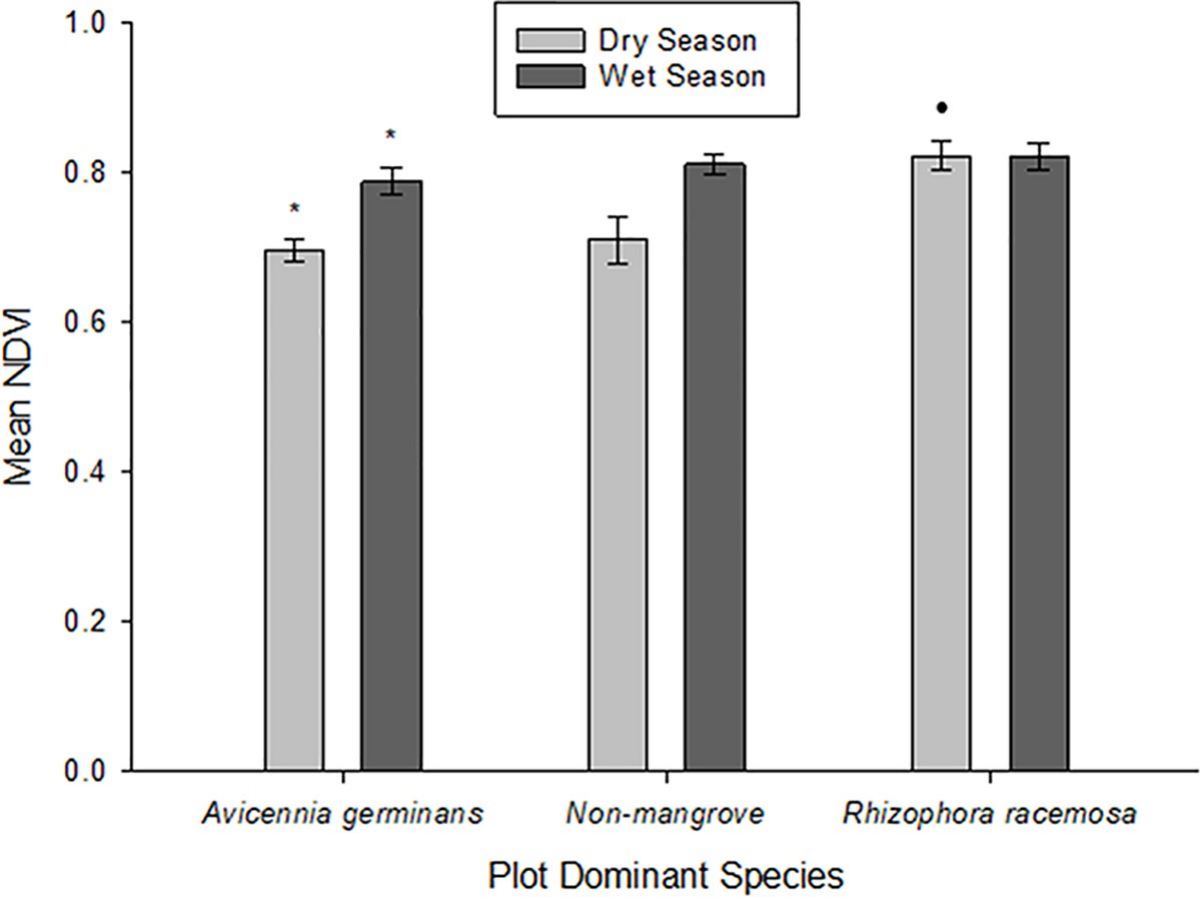
Mean NDVI values for three dominant (90% of overstory biomass) tree species in the Cabuyal estuary. Measurements derived from 10cm/pixel resolution UAS imagery taken September through November 2017 for wet season (n = 13) and January through February 2018 for dry season (n = 23) measurements. * represents significant difference of means for between season measurements of plot NDVI from paired t-tests. • represents significant difference of means for within season measurements of plot NDVI from one-way ANOVA and Tukey-HSD post-hoc tests. Error bars represent standard error from the mean.

Analysis of the dry season maps indicated three species were dominant in 23 plots at Cabuyal; *A*. *germinans* (n = 11), non-mangrove (n = 5), and *R*. *racemosa* (n = 8). The non-mangrove and mangrove species NDVI values were significantly different when compared with ANOVA (F = 20, df = 2,21, p < 0.05). Pairwise comparisons with Tukey HSD post-hoc did reveal that non-mangrove did not differ from *A*. *germinans* (p = 0.831), while *R*. *racemose* differed from non-mangrove and *A*. *germinans* (p < 0.001, p < 0.001, respectively; Fig 5). Within wet season maps, three species were determined to be dominant in 13 plots at Cabuyal; *A*. *germinans* (n = 6), *non-mangrove* (n = 3), *and R*. *racemosa* (n = 4). One-way ANOVA with Tukey HSD post-hoc pairwise comparisons showed no statistically significant difference between non-mangrove, *A*. *germinans*, and *R*. *racemosa* mean NDVI values during the wet season (F = 0.99, df = 2,10, p = 0.403). Paired t-tests between wet and dry season measurements of dominant species’ mean NDVI values for 13 corresponding plots revealed no significant difference between wet and dry season measurements of mean NDVI values for non-mangrove (t_(2)_ = 4.07, df = 2, p = 0.055) and *R*. *racemosa* (t_(2)_ = -0.51, df = 3, p = 0.645) dominant plots. A difference was found between mean NDVI values for *A*. *germinans* between wet (mean = 0.79, SE = 0.02) and dry season (mean = 0.68, SE = 0.02) measurements (t_(2)_ = 9.31, dF = 5, p < 0.001).

## Discussion

This study is the first to accurately assess the mangrove forest structure and composition of a remote, neotropical mangrove forest using a UAS. The lack of statistically significant differences found between ground-survey and UAS-imagery derived measurements of canopy height and percent coverage for high resolution maps adds to the growing body of research indicating the usefulness and accuracy of UAS in the study of mangroves and other forest ecosystems [11, 12, 13, 14, 15, 16, 17, 24, 25, 53, 54, 55, 56].

Similar to other nearby estuaries, the mangroves of Cabuyal and Zapotillal surpassed the expected size limits for north Pacific mangroves due to the arid conditions of this region, exceeding 15 m in height [57, 58]. Maximum height for *R*. *racemosa* surpassed 25 m in Cabuyal, a value considerably taller than previously reported tree heights in the more well-developed mangroves of southern Pacific Costa Rica and on par with other mangroves of the northern Pacific coast [57, 58, 59]. The banks and freshwater channels of the forest were dominated almost exclusively by *R*. *racemosa*, while *A*. *germinans* became more dominant further from water and at higher elevations. *A*. *germinans* height began to decrease on the outer fringes of the estuary, and repressed *A*. *germinans* (< 1 m height) were observable on the exterior edge of the forest, in mud flats characterized by salt encrusting on the soil surface during the dry season. Mangrove growth is influenced by a number of environmental factors, and suppressed growth in the fringe environments and mud flats is likely due to the high soil salinity and low freshwater input, while the increased growth in the lower tidal regions is likely due to the increased freshwater input in these areas, leading to increased nutrient levels and lower salinities in this portion of the forest [60, 61, 62].

The relatively similar importance value index of *A*. *germinans* and *R*. *racemosa* in the Cabuyal estuary is unique in comparison to other mangrove estuaries in the region, which are usually dominated by either *Rhizophora* spp. or *A*. *germinans* [57, 58, 60]. However, a similar pattern was found in two mangrove stands just to the south of the Papagayo peninsula, in the Bahia Culebra [57]. In the more northern estuary of Iguanita (approximately 5 km from Cabuyal), *R*. *mangle* was found to have the greatest dominance and importance value, while *A*. *germinans* was the more dominant and important species in the Panama estuary (approximately 9 km from Cabuyal), likely due to differences in hydrodynamic conditions [57]. *R*. *racemosa* tends to zonate towards low elevations in the intertidal zone, while *A*. *germinans* propagates at the higher elevations [57, 60]. The authors suggested that this area may present a transition zone between the wetter central and more arid northern Pacific mangrove stands, and the findings presented here may lend support to that theory, extending the transition area further north to the southern reaches of the Papagayo Gulf. This could make this area an ideal spot to study the ecology and biogeography of Pacific mangroves in Costa Rica, and elsewhere. However, it is critical to point out that stems below 5 cm in diameter were not considered, and further studies are necessary to perform true comparisons between these and other mangrove stands. Furthermore, there is a large range of ecological, hydrological and physical characteristics in estuarine environments which effect mangrove species zonation and distribution, and further study of these characteristics in the Cabuyal and Zapotillal estuaries are necessary before more in-depth analyses can be run [60, 61, 62, 63].

Comparisons between the Cabuyal and Zapotillal estuaries’ mangrove forests are difficult due to the lack of mangrove plots in Zapotillal (n = 1) vs. Cabuyal (n = 21). Despite its small size, the Zapotillal estuary did show features of mangrove development that warrant further investigation and possible protection of this estuary. Fed by a small seasonal stream, the estuary is small and freshwater input and tidal flooding during the wet season, but almost no aboveground freshwater input during the dry season, and the extent of mangrove vegetation is isolated to soils adjacent to the intertidal zone and freshwater channels. *R*. *racemosa* in this estuary are located along the low intertidal zones, with *L*. *racemosa* dotted along the outward edges of these stands. Small numbers of *A*. *germinans* are localized in the more upland areas of the intertidal zone. The Zapotillal estuary contained individuals of *L*. *racemosa* that were surprisingly well-developed, with a mean DBH of 32.23 cm and mean height of 19.33 m. This is far greater and among the largest individuals for this species recorded in the region [57, 58, 60]. *L*. *racemosa* has been found growing in soil salinities up to 80‰, although it is more common at lower salinities [64]. This could explain its development in a mangrove forest that only receives a short seasonal supply of freshwater and dries out considerably before the end of February (pers. observation). As *L*. *racemosa* growth is often regulated by environmental conditions at the site, with poorer quality habitat yielding reducing growth and stunting, the presence of such large individuals could be indicative of good habitat quality despite this estuary’s small size [62, 63]. This mangrove estuary should at least be included in future inventories of north Pacific mangroves, and further studies should be undertaken to understand its assemblage, structure and ecological value.

The presence of tall (> 20 m) *R*. *racemosa*, coupled with the presence of *L*. *racemosa*, *P*. *rhizophorae*, and *C*. *erectus*, all of which are more abundant in mangroves with lower soil salinities, may be indicative of greater nutrient availability and water flow dynamics via the *Quebrada Cacao* in the Cabuyal basin [57, 64]. The presence of a manmade rock jetty at the mouth of the Cabuyal estuary (the origin of which is undocumented) is another factor which may be influencing the mangrove assemblage at this site and may make it unique in the greater context of the region. Further studies of interstitial soil salinities and changes in salinities between dry and wet seasons, and further determination of the hydrology of the Cabuyal basin are needed to determine what factors have led to the local zonation and presence of different mangrove species within the Cabuyal estuary. The characterization of these estuaries has begun to fill an important gap in the description and study of mangrove forests in this region [7].

While CHM measurements of maximum canopy heights did not statistically differ from ground-based measurements, inspection of the residual plot between these measurements reveals a general increase in predictive error at taller canopy heights due to increased underestimation. This problem has been encountered in a range of studies comparing UAS-derived CHMs to ground-based measurements [19, 23, 53, 56, 65]. The accuracy of the CHM is highly correlated to the accuracy of the DTM used in its creation and is informed by the classification of different points into ground and vegetative cover [15, 26, 46, 53, 54]. Under tall, dense canopy cover, differentiation of ground points from vegetation points is a commonly reported problem in studies creating DTMs from UAS imagery alone [19, 23, 55]. Red mangroves (*Rhizophora* spp.) also tend to grow broad, irregular canopies with significant fine scale heterogeneity [66], which could increase the difficulties in accurately creating point clouds of tall red mangrove canopies, as their fine-scale architecture would be difficult to map [67]. The 10 cm spatial resolution imagery may have mapped more of the intra-crown architecture seen at greater detail inaccurately, while the 100 cm resolution imagery may have mapped more of the general crown architecture [66, 68]. This could explain the observed increase in accuracy of the coarser 100cm CHM over the finer scale 10cm CHM in determining mean canopy height. Future studies should increase flight altitude to ensure a resolution similar to 100cm per pixel, which would simultaneously increase CHM accuracy and battery efficiency during flights.

Differences between ground, 10 cm and 100 cm resolution measurements of percent canopy cover of plots had a difference between medians of 1.79% and 4.61% respectively, values similar to those found in other studies using remote sensing measurements to predict canopy cover [69, 70, 71]. Zahawi et al. (2015) [72] found an RMSE value of 12.15 between canopy cover estimates from spherical densiometer and a UAS derived measure of canopy openness in a tropical forest secondary forest in southern Costa Rica, comparable to the RMSE value seen here. Residual plot analysis reveals a pattern of increasing CCM performance at higher levels of canopy coverage. This could be due to greater contributions of understory or soil to the mean NDVI value of the plot in areas with lower canopy coverage. Carreiras et al. (2006) [71] found that the contribution of understory and soil to overall reflectance increases as canopy cover decreases, and the overall plot reflection decreases as canopy cover increases. At low canopy densities, the contribution of non-canopy reflectance therefore increases, decreasing the predictive capability of NDVI to determine canopy cover [71].

The significant difference in dry season mean NDVI values between *R*. *racemosa* dominant plots and both non-mangrove and *A*. *germinans* plots could be due to several factors. NDVI tends to saturate at greater canopy density, this could have biased higher measurements towards the taller, denser canopies of *R*. *racemosa* [73, 74, 75]. This could also explain the lack of significant difference between this group and non-mangrove wet season measurements, as this is when the non-mangrove tropical dry forest foliage would be present, and *A*. *germinans* would be experiencing less plant stress and an increase in photosynthetic capability [76, 77, 78]. The sample size (n = 3) of non-mangrove plots may have been too small to provide an adequate measure for statistical robustness in this study however. For *A*. *germinans*, photosynthetic activity has been found to significantly decrease between times of rain and drought in Venezuelan mangrove forests [77], and leaf fall has been found to increase in *A*. *germinans* during the peak of the dry season in mangrove forests in Mexico [78]. As NDVI is sensitive to both changes in photosynthetic ability and canopy cover, changes in the phenology of this species could be responsible for the changes seen in NDVI between seasons [75, 79]. Furthermore, Flores-de-Santiago et al. (2012) [80] found that in healthy *R*. *mangle* trees in Pacific Mexico, there was no significant change in upper canopy chlorophyll *a* content and leaf area index between dry and wet seasons, and a significant increase in leaf area in healthy *A*. *germinans* leaves in the wet season, though no seasonal changes in leaf area index were observed. Poor condition *A*. *germinans* also showed an increase in leaf length in the lower canopies in the wet season, and an increase in chlorophyll *a* content in the wet season. Although Flores-de-Santiago et al. (2013) [81] found NDVI to be a poor estimator of this chlorophyll *a* content, NDVI has been shown to be a good indicator of mangrove health and structural parameters in a variety of remote sensing studies from mangrove forests world-wide [82, 83, 84]. It is important to note however that only limited wet season imagery could be acquired due to the mechanical failure which occurred in November 2019. This lack of coverage could have led to a potential sampling bias in the analysis, as only a limited portion of the Cabuyal estuary could be covered. While this was accounted for in the analysis by only comparing plots that were sampled in both the wet and dry seasons, care should still be taken in interpreting the results from this study. Additionally, due to time and funding constraints, only 10 cm/pixel resolution maps were created and analyzed or this portion of the study. While this does not affect the results of the 10cm/pixel resolution analysis, running a 100cm/pixel resolution analysis would allow for a more comprehensive assessment of the utility of UAS-borne NDVI imagery for species delineation in mangrove forests, and should be undertaken in future studies. NDVI measurements can be influenced by differences in soil brightness, soil reflectivity, water absorption, canopy complexity, and path radiance, which can all affect the measurement of NDVI from imagery not calibrated with radiometric calibration images or targets [85, 86, 87, 88]. While information from the incident light sensor does correct for changes in light intensity and sun angle between flights, allowing for more accurate comparisons of NDVI measurements taken on different days, there is no full substitute for true radiometric calibration from ground-based targets. We attempted to partially correct for this by using a wide variety of land cover classes with multiple reflectance values during the creation of the SVM, but future studies should employ radiometric calibration targets and equations for atmospheric corrections when using NDVI imagery to reduce these possible effects [88, 89, 90].
